# Metastatic neuroendocrine carcinoma presenting as multifocal liver lesions with elevated alpha‐fetoprotein

**DOI:** 10.1002/ccr3.1956

**Published:** 2018-12-16

**Authors:** Lindsay A. Sobotka, Timothy Hake, Crystal Kelly, Luay Mousa

**Affiliations:** ^1^ Department of Internal Medicine The Ohio State University Wexner Medical Center Columbus Ohio; ^2^ Department of Hematology The Ohio State Wexner Medical Center Columbus Ohio

**Keywords:** alpha‐fetoprotein, hepatocellular carcinoma, liver lesions, neuroendocrine carcinoma

## Abstract

Significant elevations in alpha‐fetoprotein should raise suspicion for hepatocellular carcinoma as malignancies with metastasis to the liver can elevate the alpha‐fetoprotein level but typically <300 ng/mL. Diagnosis should be confirmed with typical characteristics of hepatocellular carcinoma on imaging and or liver biopsy to confirm diagnosis.

## 1 INTRODUCTION

Approximately 5.86 per 100 000 patients are diagnosed with neuroendocrine tumors (NET) yearly and the prevalence has been increasing since the 1970s.^1^, ^2^ The primary origin of NET is typically a gastroenteropancreatic site followed by the lung.^2^ Tumor markers associated with NET include 5‐hydroxyindoleacetic acid (5‐HIAA), gastrin, insulin, glucagon and vasoactive intestinal polypeptide depending on the primary tumor site of origin.^3^

Alpha‐fetoprotein (AFP) is a well‐established tumor marker in hepatocellular carcinoma (HCC) and yolk sac tumors.^4^, ^5^ There are rare clinical case reports describing NET producing elevation in AFP, some of which lack liver metastasis.^6^, ^7^ One study evaluated using AFP as a tumor marker in NET to determine survival; however, the number of patients with elevated AFP was limited and the average AFP level was less than 300 ng/mL.^8^ We present a case of NET with liver involvement with an AFP elevated >8000 ng/mL. To our knowledge, this is one of the first case reports of a NET causing a marked elevation of AFP.

## 2 CASE REPORT

**Figure 1:**
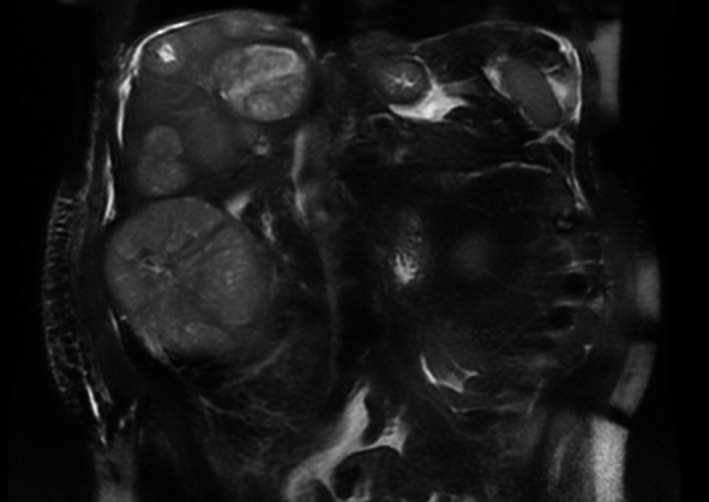
Multifocal Liver Lesions on MRI Abdomen with and without Contrast, T2 Weighted Image

**Figure 2:**
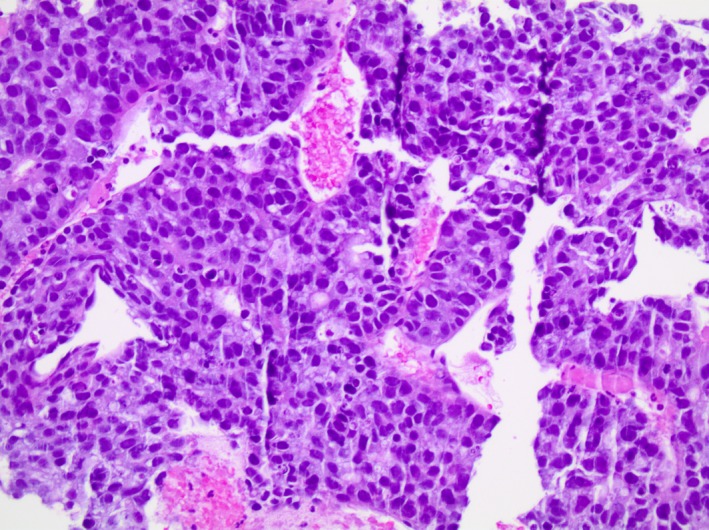
Hematoxylin and Eosin (H&E) stained histologic sections (200X), demonstrate a homogenous, population of cells with a round, uniform nuclei and a finely granular cytoplasm in a nested and trabecular patterns. Multiple foci of necrosis and occasional mitoses are conspicuous

A 77‐year‐old male with a past medical history of coronary artery disease status post coronary artery bypass grafting, hypertension, chronic obstructive pulmonary disease, diabetes mellitus type 2, and cerebrovascular accident presented to a local hospital with acute abdominal pain and bloating. A computed tomography (CT) scan of the patient's abdomen and pelvis was performed and showed intraabdominal bleed and multifocal liver lesions. Initial complete blood count (CBC) revealed a hemoglobin of 7 g/dL and he was transfused one unit of packed red blood cells prior to transfer to our institution. On arrival, CT angiogram of the abdomen and pelvis showed multiple dense, heterogeneous masses throughout the liver with associated perihepatic and intraperitoneal hemorrhage and areas of tumor blush were noted but no extravasation was seen to suggest active hemorrhage. There was also multiple enlarged periportal and upper mesenteric lymph nodes, likely representing metastatic adenopathy. There were no lesions present on the pancreas. CT chest was obtained and showed no evidence of intrathoracic metastatic disease. Initial blood work revealed normal liver function tests, appropriate response in hemoglobin to transfusion and negative viral hepatitis panel. Tumor markers revealed AFP elevation to 8705 ng/mL, normal Carcinoembryonic Antigen and Cancer Antigen 19‐9. Magnetic Resonance Imaging (MRI) of the abdomen and pelvis showed multiple lesions throughout the liver with targetoid appearance. There was no evidence of cirrhosis and these lesions did not have imaging characteristics of typical HCC (Figure 1). It was suspected the multifocal liver lesions were HCC given the elevated AFP. However, the MRI was not consistent with HCC and a liver biopsy was obtained. Pathology results were consistent with poorly differentiated, large cell‐type neuroendocrine carcinoma with metastatic disease to the liver (Figure 2). The patient had an unremarkable colonoscopy and esophagogastroduodenoscopy six months prior to presentation therefore, it was suspected the primary origin of NET was in the small bowel. Regarding the intraabdominal bleed noted on initial CT scan, this remained stable on repeat scans and surgery recommended conservative management. The planned chemotherapy regimen will be Carboplatin and Etoposide.

## 3 DISCUSSION AND CONCLUSIONS

We present a rare case of a NET with metastatic disease to the liver presenting with an AFP of 8705 ng/mL. While elevation in AFP to this extent has been noted in other malignancies with metastatic disease to the liver, this has infrequently been reported in NET with tumor burden in the liver.^8^, ^9^

Significant elevation in AFP >300 ng/mL is highly suggestive of HCC especially in the setting of liver lesions.^10^ Given the diagnosis of HCC cannot be made based off an AFP value alone, further imaging with CT or MRI with contrast must be completed. If imaging does have characteristic features of HCC based on arterial phase hypervascularity and washout and underlying cirrhosis or chronic hepatitis B, the diagnosis can be made without tissue biopsy.^11^, ^12^ As noted in this case, despite the significantly elevated AFP value and multifocal liver lesions, imaging was not suggestive of HCC and there was no evidence of underlying cirrhosis. Liver biopsy was then obtained in order to determine etiology of liver mass.^13^ Pathology was consistent with NET. While tumor makers are helpful in diagnosis of malignancy and monitoring treatment, it is crucial to keep a broad differential and obtain tissue to make a definitive diagnosis given the sensitivity and specificity of certain tumor markers.^14^

The utilization of AFP in NET is poorly understood at this point. Multiple case reports have cited elevated values of AFP in NET of different primary locations and several cases did not have liver metastasis.^6^, ^7^ Currently there are minimal studies to evaluate the ability to use AFP in the diagnosis or treatment monitoring of NET and therefore further research should be completed in order to determine possible utility of monitoring AFP in patients with NET.

## CONFLICT OF INTEREST

None declared.

## AUTHOR CONTRIBUTIONS

LS: Drafted the manuscript, edited for scientific merit and approved final draft. TH: Drafted the manuscript, edited for scientific merit and approved final draft. CK: Drafted the manuscript, edited for scientific merit and approved final draft. LM: Drafted the manuscript, edited for scientific merit and approved final draft.
